# Cannabis and children: risk mitigation strategies for edibles

**DOI:** 10.3389/fpsyt.2024.1285784

**Published:** 2024-02-06

**Authors:** Cathy Conerney, Fabian Steinmetz, James Wakefield, Sam Loveridge

**Affiliations:** ^1^ Europe, Middle East & Africa (EMEA) Toxicology Team, Delphic HSE Solutions Ltd., Camberley, United Kingdom; ^2^ Technical Services, Delphic HSE (Europe) B.V., Schiphol, Netherlands; ^3^ Technical Services, Delphic HSE Solutions (Hong Kong) Ltd., Hong Kong, Hong Kong SAR, China

**Keywords:** cannabis, risk mitigation, toxicology, packaging, child prevention, edibles, child proofing

## Abstract

In the era of (re)legalisation of medicinal and recreational cannabis, accidental and intentional exposure to edibles, cannabis-infused food products, has increased substantially. However, there is particular concern regarding younger age groups. Most concerning is the increase in hospitalisations. According to a study by Myran et al. (1), provinces in Canada, where the sale of edibles is permitted, saw an increase in paediatric poisonings due to unintentional consumption of edibles. Similar trends have been observed in “legalised states” in the US, such as Colorado (2). The impact of using cannabis at an early age, but particularly the impact of accidental exposure to high THC quantities, may have negative mental or physical health outcomes. Whilst regulatory restrictions vary significantly from one legalised region to another, it is difficult to identify a best practice. The aim of this study is to identify and discuss new and existing risk mitigation strategies to give guidance to policymakers. Furthermore, practical aspects, such as compliance (e.g. audits by authorities), are discussed. It is noted that edibles have been around much longer than recent political attempts to regulate them.

## Background

### (Re)legalisation of medicinal and recreational cannabis

Around the world, most countries attempt to abide by the United Nations (UN) Single Convention on Narcotic Drugs 1961, the Convention on Psychotropic Substances 1971 and the Convention Against Illicit Traffic in Narcotic Drugs and Psychotropic Substances 1988 (3). These international drug treaties encompass the implementation of legislations addressing legal use and the illegal drug trade. However, when it comes to cannabis, some territories, or states within countries, have their own drug laws or interpretations, which may appear to be less restrictive.

In 2020, the UN re-scheduled cannabis (4) from Schedule IV (drugs considered to be the most dangerous, with limited medicinal or therapeutic value, e.g., heroin) to a Schedule I narcotic (drugs with addictive properties but with identified potential for medicinal or therapeutic uses). This places cannabis in the same scheduling as methadone, morphine, and oxycodone (5–7).

Some of the basis for its re-classification was partly due to the growing evidence on the significant health benefits for children, particularly with certain forms of epilepsy and autism (6), as well as easing the symptoms and side effects in those undergoing cancer treatment (7). Part of this re-classification was done in the hope to give scientists and authorities more freedom in investigating its potential benefits, whilst also allowing more strict control on how it is produced and distributed. By taking back control over such medicinal products, it gives regulators more confidence in the safety of cannabis products reaching the market, compared to products obtained illicitly in an uncontrolled market.

Countries such as Canada (8), Uruguay (9) and several states in the USA (10) have also legalised recreational use of cannabis. This list is expected to grow as the weight of positive scientific data becomes known alongside ongoing legalisation (11). Some countries have decriminalised the possession of small quantities of cannabis for personal use, e.g., in Spain, Portugal and the Netherlands; when found in possession by law enforcement, most offenders are given either a verbal or written warning, a small fine and/or a counselling offer (12).

The re-classification of cannabis also saw the World Health Organisation (WHO) suggest that cannabidiol (CBD) with less than 2% tetrahydrocannabinol (THC) should not be subjected to drug controls (4), however this suggestion was rejected by several member states. The United Nations Commission on Narcotic Drugs (CND) instead voted in favour to add a footnote that a THC level < 0.2% would not be subjected to controls (13). In some cases, the basis for its rejection was due to CBD itself not being currently controlled under most drug laws. Presently, the European Commission, via the Scientific Committee on Consumer Safety (SCCS) (14), has initiated a call for data on the safety of CBD in cosmetics. The UK Home Office has also created guidance on the use of CBD in products coming into the UK (15). CBD has seen a dramatic rise in demand for inclusion into consumer goods, most likely due to current marketing trends. The market size of CBD consumer products is expected to grow at a rate of 16.2% from 2023 to 2030 (16).

Although there are more countries considering decriminalisation, there are still regions in the world where possession is still considered highly illegal and is treated the same as drugs currently under Schedule IV. This is often the case in the Middle East and areas of Africa and Asia. If found guilty of trafficking the drug, some cases result in corporal punishment, which is seen in places such as Egypt (17), Malaysia (18) and Singapore (19), and are even punishable by death.

Around Europe, a number of shop fronts that sell cannabis products for non-medicinal purposes, such as coffeeshops, can exist. In the Netherlands, each coffeeshop must be licensed by the municipality they are in; however, almost two thirds of the municipalities in the country do not permit such licenses. Consumption of cannabis for recreational uses is permitted but they must adhere to restrictions that have been set up by the public prosecutor (11). Part of these restrictions include limiting who can visit such coffee shops, with only residents of the Netherlands permitted to do so, and coffee shop owners must ensure all visitors are above 18 years of age and have a valid residence permit or are present on the municipality register (20).

## Hospitalisation rates in younger populations

According to a study by Myran et al. (1), in Canada, where the sale of cannabis-infused food products, so called “edibles”, has been permitted, the region experienced an increase in paediatric poisonings due to unintentional consumption of such edibles. Hospitalisations were slightly higher in males at < 54%, while the average age of those admitted ranged from 3-4 years. Additionally, most hospitalisations covered in the study period occurred during the later legalisation period (2020-21), compared to the pre-legalisation (2015-18) and earlier legalised study period (2018-19).

Similar trends have been observed in legalised states in the US, such as Colorado (2). Medical uses have been allowed in the state since 2001, but in 2014 a public health intervention was passed. In 2017, children hospital visits doubled, and poison control centre calls increased by 50%. The median age of children admitted was 3 years, with 58% being female. Most cases were linked with ingestion, with edibles being the second highest product category after the dried plant.

In the adolescent population (15–19-year-olds), where a previous decline in cannabis use was noted before legalisation, this may now be starting to rise once more due to the increased accessibility of the drug (21). This is somewhat reflected in the findings by Myran et al. (1), where paediatric hospitalisations increased substantially when comparing the pre- and post-legalisation periods. The rate of poisonings per 1000 went from 57.42 in exposed areas, to 318.04 post-legalisation (1). Canada also has one of the highest rates of cannabis use amongst young people in the world, however a small decline in the 12-month use prevalence has been recognised in 16–19-year-olds (from 41% in 2017 to 37% in 2022) (22, 23).

Similar scenarios have also been observed within Europe, particularly in France, where it has one of the highest recorded cannabis uses on the continent even without legalisation. A study in Marseille by Mehamha et al. (24) noted that cannabis poisoning amongst toddlers (mean age 17 months) was becoming more frequent, including a rise in severity (24, 25).

Conversely, however, some studies note a decrease in cannabis use post-legalisation. A study by Midgette & Reuter (26) detailed that in Washington State, 30-day cannabis use after legalisation in fact decreased 22% and 12.7% in grade 8 and grade 10 students respectively, but no effect was noted in grade 12. In Uruguay, although an initial spike in use was noted in 18–21-year-olds post-legalisation, usage rates returned to pre-legalisation levels according to Rivera-Aguirre et al. (27).

Based on trends seen in the US, the most common forms of cannabis consumption in young people other than traditional smoking include vaping (28), which in itself has seen massive growth in recent years, as well as the consumption of edibles (29). However, the consumption of edibles, considered safe by some due to absence of inhalation of cannabis smoke (30), can still result in cannabis intoxication, particularly in young children (31).

**Figure 1:**
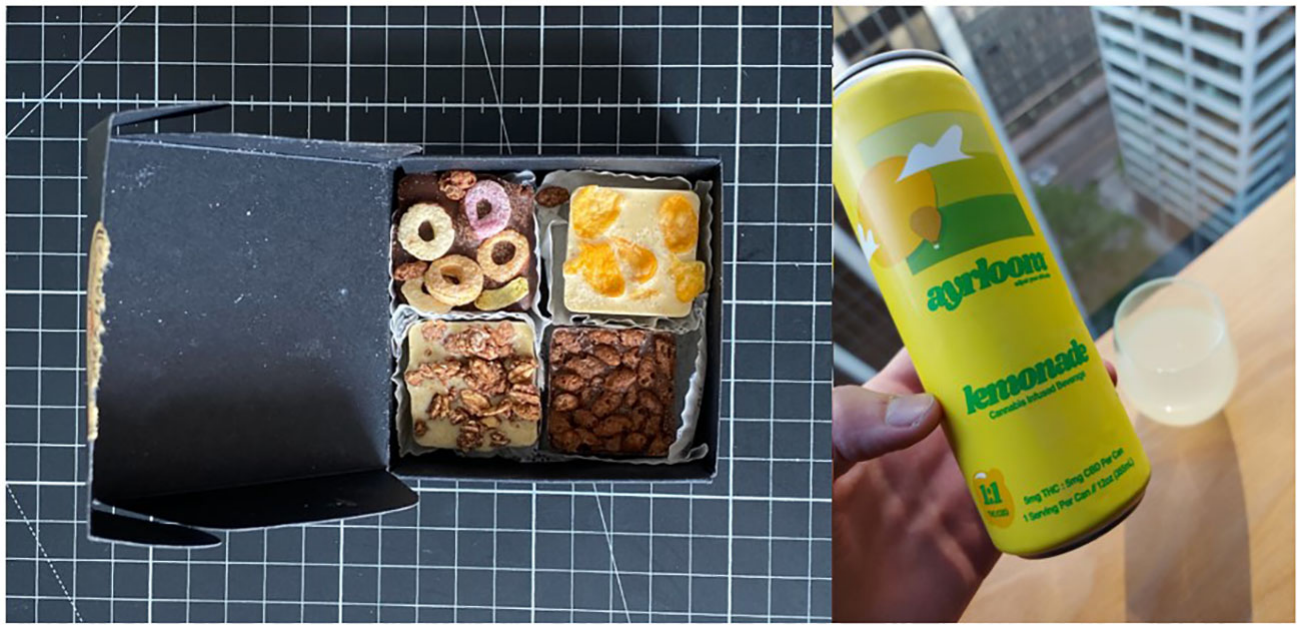
Examples of ‘hash brownies’ & cannabis infused beverages. Images courtesy of Joe & Constantin Koch (left) and Herr Greenrush (right).

The most common form of edibles is usually presented as baked goods, such as brownies, often nicknamed ‘hash brownies’ (see **Figure 1**), notably due to the need for a fatty carrier (e.g., butter) and the ease of manufacture. Other examples include THC-infused chocolates, gummies and, due to advances in formulation technologies, beverages. Similar products with CBD instead of THC exist too, however, due to THC’s intoxicating properties, the focus of this manuscript lies with edibles containing significant amounts of THC.

One of the reasons for the observed rise in the consumption of edibles by children is based on the packaging, and appeal thereof. Many examples available online play on imagery like well-known brands, including colourful artwork and marketing, and to the untrained eye, may look very much like a standard candy or baked good that is already on market. This is known in some cases as “attractive nuisance” (32) – a hazardous condition or object a person/company manufactures that is attractive to children, in this case packaging edibles that look appealing to the younger population.

A similar case of this “nuisance” can be seen in the food industry, particular in children’s cereal products. Not only does the colourful artwork and cartoon appeal to a child’s interest, in some cases free toys or activities are included in or on the box. In 2020, this nuisance was addressed by Mexican authorities who enacted laws for warning labels to be placed on food packaging which contain excess sugar, calories, sodium, fats, as well as caffeine. In addition to these warning labels, restrictions were also placed on the use of cartoon characters. If a product contains one of the excess warning labels, then it is not permitted to incorporate the use of cartoons. In most cases, over-labelling to cover such artwork has been implemented, versus printing cartoon-free packaging (33).

Innovation has also played a part in this increased consumption, as the palatability of edibles has also improved and become more attractive (34), which entices children to continue eating the substance without knowing or recognising the distinctive aroma that is typically associated with cannabis. Because of this, children, as well as adults, may continue to consume a larger than intended quantity due to the appealing taste, unaware that they are becoming intoxicated over time until adverse effects begin to take place.

### What are the symptoms of cannabis intoxication?

Cannabis has physical and mental effects that can begin within minutes, but may take hours to become noticeable, particularly with edibles. This is dependent on the preparation method, route of administration and concentration of source material. The physical effects include an increase in heart rate, peripheral vasodilatation, conjunctival suffusion, bronchodilatation, dryness of the mouth and, in large doses, tremor, ataxia, nystagmus, nausea, and vomiting (35). Mental effects include euphoria, spacial and time distortions, increased intensity of senses and motor impairment. This can include negative psychological reactions ranging from panic to depression (33).

Inhaled doses equivalent to 2 to 3 mg THC, and ingested amounts of 5 to 20 mg THC can result in reduction of attention span, memory (both short- and long-term) and executive functioning. Doses above 7.5 mg/m^2^ THC as inhaled (body surface area-based dosing, equivalent to 0.2027 mg/kg or 12 mg/day on average) in adults, and 5 to 300 mg THC ingested by children can produce severe symptoms including low blood pressure, panic, anxiety, uncontrollable muscle spasms, delirium, respiratory depression and general ataxia. In children, signs of life-threatening toxicity include lethargy and/or heightened muscle movement. Although acute toxicity is uncommon in adult patients, they are likely to have severe or prolonged vomiting, behavioural problems and/or a medical emergency due to trouble breathing (36).

Adults typically present with psychiatric disorders in cases of cannabis overdose, as well as respiratory disorders due to smoke inhalation. These symptoms can develop into seizures, tachycardia, respiratory depression, and coma (37). Adverse effects in children are typically less known, but due to the rise in hospitalisation of children in states where cannabis has become legalised, more health information is becoming available. In some cases, children typically present with impaired consciousness and respiratory disorders, rather than psychiatric symptoms which are more prevalent in adolescents and adults (38). Admissions data is also starting to reflect that paediatric patients are experiencing more severe symptoms with longer hospital stays (39).

Other long-term effects are still relatively unknown; some studies suggest long-term THC use, or use from an early age, is associated with the onset of psychosis and other neurological disorders later in life. However, the scientific community is still puzzled with addressing directionality (40) – is cannabis use leading to psychopathologies or are psychopathologies leading to cannabis use? Decreased level of intelligence, as well as decreases in attention span and memory capabilities have also been noted in heavy users, although most findings are reversible (41, 42). Use of cannabis during pregnancy is also speculated to contribute to negative, long-term health effects in the offspring (43).

A recent meta-analysis conducted by Allaf et al. (44) concluded that decriminalisation saw a rise in acute poisonings, particularly in the USA. However, it is worth noting that this region has not yet fully legalised cannabis use, as policies are different from state to state. Meta-analysis in countries, such as Canada or decriminalised states, would be considered more relevant (45).

## Risk mitigation

### Packaging and labelling of edibles

For cannabis products, child-resistant packaging is necessary to prevent children from using a product that may lead to unwanted, adverse effects. Packaging types are typically characterised into ‘reclosable’ and ‘non-reclosable’ containers, the difference being that once opened, the latter cannot be resealed or sufficiently closed to provide a degree of security from children. These closures are defined by the international standards of BS EN ISO 8317:2004 and BS EN 14375:2003, respectively (46, 47). Examples of reclosable packs include “push and turn”, or “squeeze and turn” caps, often utilised for pharmaceutical containers. Non-reclosable pack types include blister packs often seen for holding tablets or capsules. To be certified as child-resistant, the packaging must comply with one of the packaging standards, including panel testing in children where 85% must fail to open the packs before demonstration, and 80% after demonstration (48, 49). The child-resistant packaging of edibles is therefore one method of risk mitigation to prevent and deter children from accidental, or intentional, ingestion.

**Figure 2:**
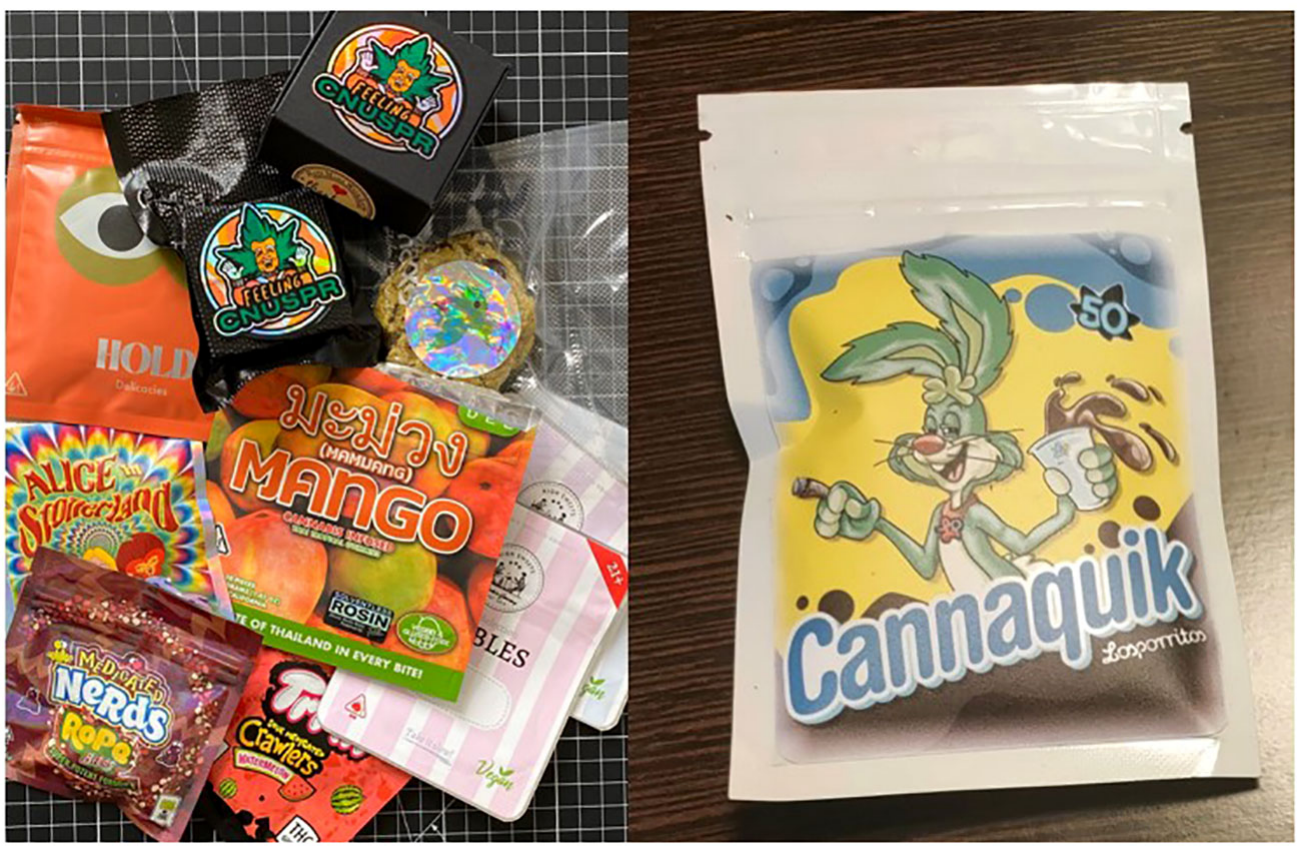
Examples of “edibles” with so-called ‘attractive nuisance’, reflecting cartoon imagery and well-known consumer brands including ‘Nesquik’. Images courtesy of Joe & Constantin Koch (left) and ParaSid (right).

**Figure 3:**
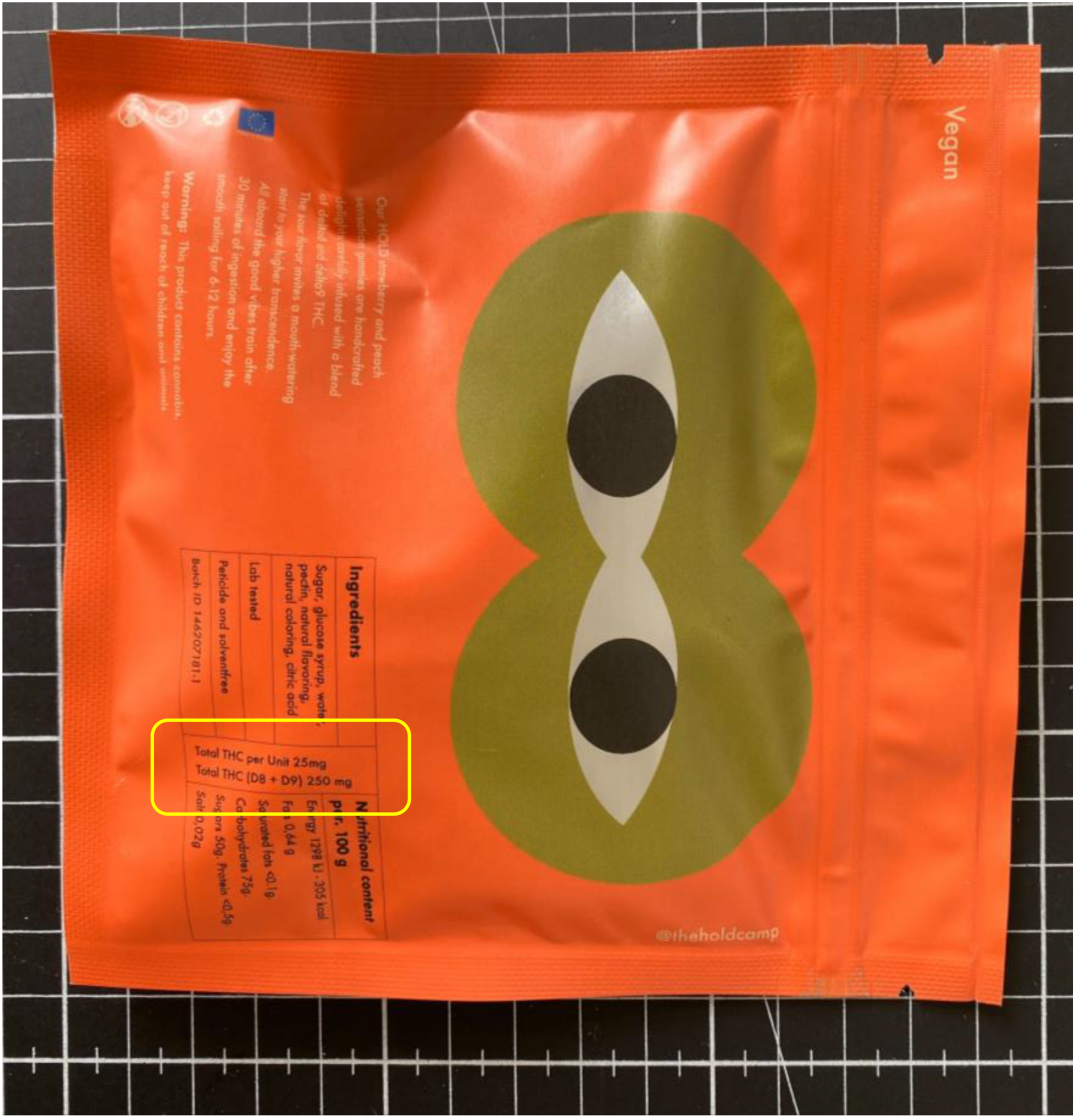
Example of dosing supplied on edible products (in yellow). Image courtesy of Joe & Constantin Koch.

Along with traditional child-resistant packaging, innovation in the packaging industry has also seen the development of child resistant zipper bags or pouches, which include locking “press and seal” zipper (50) technology (see **Figures 2**, **3**), also produced in accordance with child resistant packaging laws in the USA.

In Canada, the distribution of cannabis products is regulated under the Cannabis Act 2018 (8) with specific requirements on packaging and labelling, as well as limits on THC content and controls on other ingredients including caffeine, sugars, and nicotine. Packaging must be child-resistant, in plain appearance (i.e., depictions of persons, characters or animals, images that evoke a sense of positive emotion such as excitement should not be present), as well as including certain health warnings. The overall product must not be appealing to children, limiting the “attractive nuisance”.

Similar restrictions also exist in some US states where recreational cannabis has been legalised e.g., Colorado, Washington and Alaska (51). However, such guidance specifically for cannabis products is not enforced in other regions where its use has been legalised, and hence the probability of children and adolescents buying and consuming products that look like regular foodstuffs is heightened.

A study by Ventresca & Elliot (2022) (52) recruited participants in Canada to assess current approved cannabis packaging in the region to determine what additional information they would like to see on cannabis products. Although child-resistant packaging was suggested, a notable suggestion was the desire to know the standardised THC unit, consumption instructions and unit-dose packaging. Health Canada stipulates no more than 10 mg of THC can be present in edibles, yet products purchased outside the region, or manufactured illegally, may contain unknown amounts of the psychoactive, as well unquantifiable amounts of other, potentially harmful cannabinoids.

By knowing the actual dose of THC or total cannabinoid content in products, consumers are more aware of their acceptable daily dose, and from this can determine a tolerable limit. This is especially important in cases of parents dosing children for the sake of easing the symptoms of illness (which is still considered illegal in most legalised states). By not knowing the cannabinoid content in products bought at various times of the year or from various locations, parents could unknowingly be putting their children at an even higher risk when the dosing is not accurately labelled or quantified.

### Dosing and ingredient identification

Although the implementation of child resistant packaging is one part of helping solve this issue, the amount of THC and other cannabinoids incorporated into products should also be indicated clearly to ensure consumers are well-informed, and in potential cases of intoxication or overdose, health professionals can more accurately identify adequate treatment options.

THC is hardly soluble in water (53). Whilst traditional baked goods use either hashish or cannabis butter/oil (i.e., butter or vegetable oil infused with herbal cannabis) (54), there is a trend to use high potency extracts, such as decarboxylated “butane honey oil” (a potent extract often referred to as BHO) or THC distillates with a purity >95%. With this technology it is possible to add 60 mg THC (and potentially more) into a single piece of candy gum (55). Recently, sugar (cf. THC syrup) and microfluidizer have been used to increase applications and solubility in water, e.g., for mixing in beverages (56, 57). Some of these products lack the typical cannabis flavour and/or contain extremely high THC quantities. Both aspects, particularly in combination, can increase the risk for either accidental exposure or underestimation of the potency. However, heavy users may desire to take oral THC doses of >100 mg. These include THC-rich extracts in a syringe where people can dose an accompanied plain brownie to their liking (58).

A separate study by Vandrey et al. (59) reported that out of 75 products tested that were purchased within the US, over 60% had less THC than labelled, while 23% had more THC than labelled, and in some cases no THC at all. A similar study by the US FDA (60) noted that out of 102 CBD products, 45% of products contained within 20% of the amount indicated, and 37% contained more than 120% of the indicated CBD. Within these, 49% of products contained THC when it was not indicated.

### Informing beyond the product packaging

**Figure 4:**
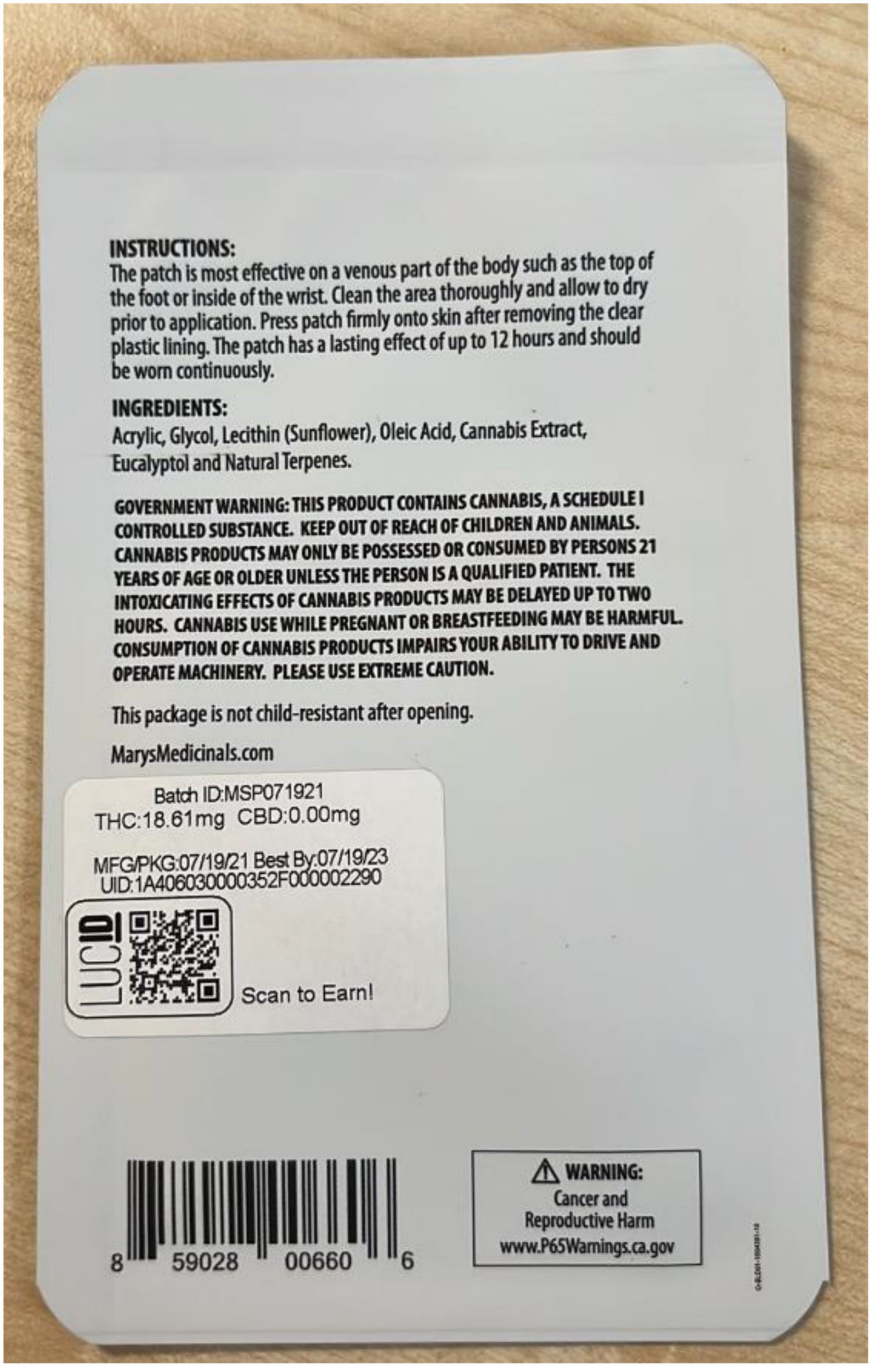
Example of products with lengthy warning and instructions for use, THC & CBD dosing, and the presence of a California Prop65 warning label (bottom right). Image courtesy of Constantin Koch.

The communication of the risks of edible cannabis products must go beyond the product packaging itself, particularly as younger children are more likely to consume something that looks like food without consulting the label (see **Figure 4**). This issue is also seen in the cosmetics industry, particularly in cases of bath products such as bath bombs that resemble foodstuffs such as cupcakes or donuts, in terms of size, shape and even smell. Such cases are regulated under directives in both the EU (61) and UK (62), whereby products resembling food which are not edible must not be placed on the market, or risk being recalled, and significant fines being issued to the manufacturer.

A key strategy is then to reduce the potential for intended users to leave the product in reach of children by informing them of the dangers, like other consumer goods with intrinsic hazards such as alcohol, nicotine, medication, or cleaning goods. The biggest, positive impact is expected when the message is spread over multiple platforms and from different bodies, including government (63), non-government organisations (NGOs) (64) and the companies selling the products themselves. Nicotine-containing liquids, a recent product category where child poisoning also increased rapidly alongside increased sales, had a combined risk informing strategy in the USA via online websites, TV adverts and presentations at schools. This is having an impact on hospitalisation rates, although confounding factors may also be at play (65).

Prevention controls in schools is also an approach that has been taken by a number of states in the US where cannabis has been legalised. A case study in California, which was the first state to legalise its use for medicinal use, as well as one of the first for non-medicinal use, reported that numerous prevention and awareness classes are now taught to 7-11^th^ graders (12–17-year-olds). Project ALERT is a taught science course on how to resist the use of drugs and alcohol, with similar courses such as Project TND (Towards No Drug Abuse) and CAPT (Cannabis Awareness and Prevention Toolkit) also being implemented in the state (66).

### Control and enforcement

One of the easiest ways to reduce the likelihood of exposure to particularly harmful products is to control the supply to the market. This can be done by imposing regulations with appropriate restrictions on who can manufacture, who can sell and who can buy cannabis-infused food products. Batch-specific specifications and traceability from plant to product, like that already in place for food products, such as for meat and poultry, could be used to provide consumers and regulators with a level of transparency. This should come along with typical consumer good practices, such as audits by appropriate authorities, mystery shopping, analyses of random samples and, if necessary, issuing penalties, forcing market withdrawals, or closing facilities and shops (after multiple or very severe violations). In cases of frequent or serious adverse events caused by a single products or product ranges, relevant batches should be identified and traced back to the manufacturer. Beyond that, authorities should check appropriate customer communication, for instance, that no health claims are made. Additionally, authorities monitoring the overall quality of products on sale could identify new trends and harm potentials arising. However, it is important that regulations and penalties are set and enforced in a sufficiently tolerant way to allow for a legal, health-oriented market to develop as alternative to an unregulated market with harmful products and harmful law enforcement towards consumers.

## Conclusion

Although legislation is a necessary step to overcome the harms created by current laws on cannabis, access to certain cannabis products, including edibles, comes with its unique challenges. As has been reported, the number of hospitalisations of children who have consumed cannabis products, either intentional or through misuse, is increasing, even in regions were legislation has already been implemented. To help reduce the risk of unintentional intoxication in children, appropriate mitigation measures must be implemented to ensure their safe use. These include child-resistant packaging and absence of imagery that contributes to ‘attractive nuisance’.

In addition, beyond the responsible storing of edibles out of children’s reach, cannabis products should be appropriately labelled in terms of THC dosing per piece and per package, inclusion of clear instructions and warnings, and advice on what to do should overdosing lead to cannabis intoxication. Manufacturers should ideally also make available batch-specific specifications, and ensure traceability of the product’s manufacturing process, as well as standard quality checking. This guidance will give policymakers a better understanding in how to manage the sale and distribution of edibles and other cannabis products. Any strategy for tackling the potential for misuse of products should look to implement continuous quality improvement. It is also important to share the successes, improvements and learning of any actions with the global community as this continues to be a rapidly developing area of interest.

## Author contributions

CC: Investigation, Resources, Visualization, Writing – original draft. FS: Conceptualization, Investigation, Resources, Visualization, Writing – review & editing. JW: Writing – review & editing. SL: Investigation, Resources, Writing – review & editing.

## References

[1] Myran DT Tanuseputro P Auger N Konikoff L Talarico R Finkelstein Y. Pediatric hospitalizations for unintentional cannabis poisonings and all-cause poisonings associated with edible cannabis product legalization and sales in Canada. JAMA Health Forum (2023) 4(1):e225041. doi: 10.1001/jamahealthforum.2022.5041 36637814 PMC9857209

[2] Wang GS Hoyte C Roosevelt G Heard K. The continued impact of marijuana legalization on unintentional pediatric exposures in Colorado. Clin Pediatr (2019) 58(1):114–6. doi: 10.1177/0009922818805206 30288992

[3] United Nations Office on Drugs and Crime (UNODC). The International Drug Control Conventions, United Nations Office Vienna (2013). Available at: https://www.unodc.org/documents/commissions/CND/Int_Drug_Control_Conventions/Ebook/The_International_Drug_Control_Conventions_E.pdf.

[4] United Nations. UN commission reclassifies cannabis, yet still considered harmful. UN News (2020). Available at: https://news.un.org/en/story/2020/12/1079132.

[5] United Nations. Conference for the Adoption of a Single Convention on Narcotic Drugs, 1961. Single Convention on Narcotic Drugs. New York, United States: US Government Printing Offic (1961). Available at: https://www.unodc.org/pdf/convention_1961_en.pdf.

[6] Riboulet-Zemouli K. High Compliance, a Lex Lata Legalization for the Non-Medical Cannabis Industry: How to Regulate Recreational Cannabis in Accordance with the Single Convention on Narcotic Drugs, 1961. DC: FAAAT editions (2022). Available at: https://papers.ssrn.com/sol3/papers.cfm?abstract_id=4057428.

[7] Jelsma M Bewley-Taylor D Blickman T Walsh J. A House of Cards, ‘High compliance’: A legally indefensible and confusing distraction (2022). Available at: https://longreads.tni.org/a-house-of-cards.

[8] Government of Canda. Cannabis Legalization and Regulation, Cannabis Act S.C. 2018, c. 16 (2019). Cannabis Act (justice.gc.ca. https://laws-lois.justice.gc.ca/eng/acts/c-24.5/

[9] Laqueur H Rivera-Aguirre A Shev A Castillo-Carniglia A Rudolph KE Ramirez J. The impact of cannabis legalization in Uruguay on adolescent cannabis use. Int J Drug Policy (2020) 80:102748. doi: 10.1016/j.drugpo.2020.102748 32388170 PMC10686048

[10] National Conference of State Legislatures (NCSL). State Medical Cannabis Laws. ncsl.org (2023). https://www.ncsl.org/health/state-medical-cannabis-laws

[11] Aran A Cayam-Rand D. Medical cannabis in children. Rambam Maimonides Med J (2020) 11(1). doi: 10.5041/RMMJ.10386 PMC7000154 32017680

[12] Dariš B Tancer Verboten M Knez Ž Ferk P. Cannabinoids in cancer treatment: Therapeutic potential and legislation. Bosnian J Basic Med Sci (2019) 19(1):14–23. doi: 10.17305/bjbms.2018.3532 PMC6387667 30172249

[13] Pardal M. Cannabis Legalization in Europe: Planning Ahead, The RAND Blog (2022). Available at: https://www.rand.org/blog/2022/11/cannabis-legalization-in-europe-planning-ahead.html.

[14] European Monitoring Centre for Drugs and Drug Addiction (EMCDDA). Cannabis policy: status and recent developments . Available at: https://www.emcdda.europa.eu/publications/topic-overviews/cannabis-policy/html_en.

[15] United Nations Commission on Narcotic Drugs (CND). Press Statement – 2 December 2020, CND Votes on recommendations for cannabis and cannabis-related substances . Available at: https://www.unodc.org/documents/commissions/CND/CND_Sessions/CND_63Reconvened/Press_statement_CND_2_December.pdf.

[16] European Commission Consultations. Call for data on ingredients used in cosmetic products (2023). Available at: https://single-market-economy.ec.europa.eu/consultations/call-data-ingredients-used-cosmetic-products-0_en.

[17] UK Home Office. Drugs and Firearms Licensing – Crime, Policing and Fire Group, ‘Factsheet – Cannabis, CBD and other cannabinoids’, Version 1.4 (2019). Available at: https://assets.publishing.service.gov.uk/government/uploads/system/uploads/attachment_data/file/825872/factsheet-cannabis-cbd-and-cannabinoids-2019.pdf.

[18] Grand View Research. Cannabidiol Market Size, Share & Trends Analysis Report by Source Type (Hemp, Marijuana), By Sales Type (B2B, B2C), By End-Use (Medical, Personal Use), By Region, and Segment Forecasts, 2023-2030 (2023). Available at: https://www.grandviewresearch.com/industry-analysis/cannabidiol-cbd-market.

[19] United Nations Office on Drugs and Crime (UNODC). Laboratory and Scientific Advice Portals, Drug Laws/Individual Lists for EGYPT . Available at: https://www.unodc.org/LSS/Country/DetailsLegalSystem?code=DLIL&country=EG.

[20] Laws of Malaysia, Act 234, Dangerous Drugs Act 1952 (Revised 1980), Incorporating latest amendment – P.U. (A) 296/2021 . Available at: https://www.pharmacy.gov.my/v2/sites/default/files/document-upload/dangerous-drugs-act-1952-act-234_4.pdf.

[21] Central Narcotics Bureau. Singapore’s Drug Laws and Enforcement Efforts, Commonly Asked Questions (2023). Available at: https://www.cnb.gov.sg/docs/default-source/educational-resources-documents/pde-info-kit-faq-(final).pdf.

[22] Government of the Netherlands. Toleration policy regarding soft drugs and coffee shops . Available at: https://www.government.nl/topics/drugs/toleration-policy-regarding-soft-drugs-and-coffee-shops (Accessed 3rd January 2024).

[23] Health Canada. Canadian Cannabis Survey 2017 (2017). Available at: https://www.Canada.ca/en/health-Canada/services/publications/drugs-health-products/canadian-cannabis-survey-2017-summary.html (Accessed 27/07/2023).

[24] Mehamha H Doudka N Minodier P Néant N Lacarelle B Solas C. Unintentional cannabis poisoning in toddlers: A one year study in Marseille. Forensic Sci Int (2021) 325:110858. doi: 10.1016/j.forsciint.2021.110858 34091410

[25] Health Canada. Canadian Cannabis Survey 2022 (2022). Available at: https://www.Canada.ca/en/health-Canada/services/drugs-medication/cannabis/research-data/canadian-cannabis-survey-2022-summary.html (Accessed 27/07/2023).

[26] Midgette G Reuter P. Has cannabis use among youth increased after changes in its legal status? A commentary on use of monitoring the future for analyses of changes in state cannabis laws. Prev Sci (2020) 21(1):137–45. doi: 10.1007/s11121-019-01068-4 PMC6960330 31792712

[27] Rivera-Aguirre A Castillo-Carniglia A Laqueur HS Rudolph KE Martins SS Ramírez J. Does recreational cannabis legalization change cannabis use patterns? Evidence from secondary school students in Uruguay. Addiction (2022) 117(11):2866–77. doi: 10.1111/add.15913 35491741

[28] Chartier C Penouil F Blanc-Brisset I Pion C Descatha A Deguigne M. Pediatric cannabis poisonings in France: more and more frequent and severe. Clin Toxicol (2021) 59(4):326–33. doi: 10.1080/15563650.2020.1806295 32840407

[29] UK Office for Health Improvement & Disparities. Research and analysis, Nicotine vaping in England: 2022 evidence update main findings (2022). Available at: https://www.gov.uk/government/publications/nicotine-vaping-in-england-2022-evidence-update/nicotine-vaping-in-england-2022-evidence-update-main-findings#:~:text=vaping%20prevalence%20among%20adults%20who,compared%20with%202.2%25%20in%202021.

[30] Chadi N Minato C Stanwick R. Cannabis vaping: Understanding the health risks of a rapidly emerging trend. Paediatr Child Health (2020) 25(Suppl 1):S16–20. doi: 10.1093/pch/pxaa016 PMC7757764 33390752

[31] Advanis Inc, Prepared for Health Canada. (2022). Available at: https://publications.gc.ca/collections/collection_2021/sc-hc/H21-312-2021-2-eng.pdf.

[32] Mahamad S Wadsworth E Rynard V Goodman S Hammond D. Availability, retail price and potency of legal and illegal cannabis in Canada after recreational cannabis legalisation. Drug Alcohol Rev (2020) 39(4):337–46. doi: 10.1111/dar.13069 32291811

[33] White M Barquera S. Mexico adopts food warning labels, why now? Health Syst Reform (2020) 6(1):e1752063. doi: 10.1080/23288604.2020.1752063 32486930

[34] MacCoun RJ Mello MM. Half-baked–the retail promotion of marijuana edibles. New Engl J Med (2015) 372(11):989–91. doi: 10.1056/NEJMp1416014 25760351

[35] Dictionary.com. Attractive Nuisance . Available at: https://www.dictionary.com/browse/attractive-nuisance.

[36] Wang GS Roosevelt G Heard K. Pediatric marijuana exposures in a medical marijuana state. JAMA Pediatr (2013) 167(7):630–3. doi: 10.1001/jamapediatrics.2013.140 23712626

[37] Hartsel JA Eades J Hickory B Makriyannis A. Chapter 53: cannabis sativa and hemp. In: Gupta RC, editor. Nutraceuticals Efficacy, Safety and Toxicity 2016 (2016). p. 735–54. https://www.researchgate.net/publication/301291128_Chapter_Title_Cannabis_sativa_and_Hemp_httpstoreelseviercomNutraceuticalsisbn-9780128021477

[38] Scott JC. Impact of adolescent cannabis use on neurocognitive and brain development. Child Adolesc Psychiatr Clinics North America (2023) 32(1):21–42. doi: 10.1016/j.chc.2022.06.002 36410904

[39] Noble MJ Hedberg K Hendrickson RG. Acute cannabis toxicity. Clin Toxicol (Phila) (2019) 57(8):735–42. doi: 10.1080/15563650.2018.1548708 30676820

[40] Turner AR Spurling BC Agrawal S. Marijuana toxicity. [Updated 2023 feb 12]. In: StatPearls. Treasure Island (FL: StatPearls Publishing (2023). Available at: https://www.ncbi.nlm.nih.gov/books/NBK430823/. 28613573

[41] Jouanjus E Leymarie F Tubery M Lapeyre-Mestre M. Cannabis-related hospitalizations: unexpected serious events identified through hospital databases. Br J Clin Pharmacol (2011) 71(5):758–65. doi: 10.1111/j.1365-2125.2010.03897.x PMC3093081 21204913

[42] Burrows K Williams JA. THC intoxication in a 16-month-old child. Paediatr Child Health (2019) 24(5):299–300. doi: 10.1093/pch/pxz015 31379428 PMC6656951

[43] Torres CA Medina-Kirchner C O’Malley KY Hart CL. Totality of the evidence suggests prenatal cannabis exposure does not lead to cognitive impairments: A systematic and critical review. Front Psychol (2020) 11:816 doi: 10.3389/fpsyg.2020.00816 32457680 PMC7225289

[44] Allaf S Lim JS Buckley NA Cairns R. The impact of cannabis legalization and decriminalization on acute poisoning: A systematic review. Addiction (2023) 118(12):2252–74. doi: 10.1016/j.puhe.2023.06.012 PMC10952774 37496145

[45] Pasman JA Verweij KJH Gerring Z Stringer S Sanchez-Roige S Treur JL. GWAS of lifetime cannabis use reveals new risk loci, genetic overlap with psychiatric traits, and a causal influence of schizophrenia. Nat Neurosci (2018) 21(9):1161–70. doi: 10.1038/s41593-018-0206-1 PMC6386176 30150663

[46] Child resistant packaging-Requirements and testing procedures for reclosable packages (2004). Available at: https://www.iso.org/standard/61650.html.

[47] -resistant non-reclosable packaging for pharmaceutical products-Requirements and testing (2003). Available at: https://www.en-standard.eu/csn-en-14375-child-resistant-non-reclosable-packaging-for-pharmaceutical-products-requirements-and-testing/.

[48] British Plastics Federation (BPF). Child Resistant Packaging . Available at: https://www.bpf.co.uk/plastipedia/applications/child-resistant-packaging.aspx#:~:text=HOW%20IS%20A%20PACK%20CERTIFIED,non%20reclosable%20packaging%20for%20medicines.

[49] British Standards Institute (BSI) Group. Child resistant packaging, A consumer’s guide to the standards for child resistant packaging . Available at: https://www.bsigroup.com/LocalFiles/en-GB/consumer-guides/resources/BSI-Consumer-Brochure-Child-Resistant-Packaging-UK-EN.pdf.

[50] DymaPak. SecureSack Child Resistant Bags . Available at: https://dymapak.com/child-resistant-bags/.

[51] Goodman S Hammond D. THC labeling on cannabis products: an experimental study of approaches for labeling THC servings on cannabis edibles. J Cannabis Res (2022) 4(1):17. doi: 10.1186/s42238-022-00124-1 35387681 PMC8988394

[52] Ventresca M Elliott C. Cannabis edibles packaging: Communicative objects in a growing market. Int J Drug Policy (2022) 103:103645. doi: 10.1016/j.drugpo.2022.103645 35276401

[53] National Center for Biotechnology Information. PubChem Compound Summary for CID 16078, Dronabinol (2023). Available at: https://pubchem.ncbi.nlm.nih.gov/compound/Dronabinol.

[54] Barrus DG Capogrossi KL Cates SC Gourdet CK Peiper NC Novak SP. Tasty THC: Promises and Challenges of Cannabis Edibles. Methods report Vol. 2016. RTI Press (2016). doi: 10.3768/rtipress.2016.op.0035.1611 PMC5260817 28127591

[55] Leafly. How to make edibles with concentrates and dabs (2022). Available at: https://www.leafly.com/learn/consume/edibles/how-to-make-edibles-cannabis-concentrates.

[56] Leafly. Recipe: How to make cannabis-infused maple syrup (2019). Available at: https://www.leafly.com/news/Canada/recipe-cannabis-infused-maple-syrup.

[57] Instrumat. Microfluidics technology to Produce water soluble CBD and THC nanoemulsions . Available at: https://instrumat.ch/ic-article/microfluidics-technology-to-produce-water-soluble-cbd-and-thc-nanoemulsions/.

[58] Bulkweed Inbox. THC Distillate Syringes – 1 mL . Available at: https://bulkweedinbox.cc/product/thc-distillate-syringes-1-ml/ (Accessed 21st August 2023).

[59] Vandrey R Raber JC Raber ME Douglass B Miller C Bonn-Miller MO. Cannabinoid dose and label accuracy in edible medical cannabis products. JAMA (2015) 313(24):2491–3. doi: 10.1001/jama.2015.6613 26103034

[60] U.S. Food and Drug Administration. Report to the U.S. House “Sampling Study of the Current Cannabidiol Marketplace to Determine the Extent That Products are Mislabeled or Adulterated Report in Response to Further Consolidated Appropriations Act, 2020. U.S. Food and Drug Administration: FDA (2020). Available at: https://hempindustrydaily.com/wp-content/uploads/2020/07/CBD-Marketplace-Sampling_RTC_FY20_Final.pdf.

[61] Council Directive 87/357/EEC of 25 June 1987 on the approximation of the laws of the Member States concerning products which, appearing to be other than they are, endanger the health or safety of consumer . Available at: https://eur-lex.europa.eu/legal-content/EN/TXT/?uri=celex%3A31987L0357.

[62] STATUTORY INSTRUMENTS 1989 No. 1291 CONSUMER PROTECTION The Food Imitations (Safety) Regulations 1989 . Available at: https://www.legislation.gov.uk/uksi/1989/1291/made#:~:text=The%20Regulations%20prohibit%20persons%20from,being%20defined%20so%20as%20to.

[63] Centres for Disease Control and Prevention (CDC). Smoking & Tobacco Use, Quick Facts on the Risks of E-cigarettes for Kids, Teens, and Young Adults . Available at: https://www.cdc.gov/tobacco/basic_information/e-cigarettes/Quick-Facts-on-the-Risks-of-E-cigarettes-for-Kids-Teens-and-Young-Adults.html (Accessed 20th August 2023).

[64] American Lung Association. What It Means to Be “Nic-Sick” (2019). Available at: https://www.lung.org/blog/nic-sick (Accessed 20th August 2023).

[65] Chang JT Wang B Chang CM Ambrose BK. National estimates of poisoning events related to liquid nicotine in young children treated in US hospital emergency departments, 2013–2017. Inj Epidemiol (2019) 6:10. doi: 10.1186/s40621-019-0188-9 Wang JT Chang B Bk Ambrose CM 31245259 PMC6582692

[66] Substance Abuse and Mental Health Services Administration (SAMHSA). Chapter 4 – examples of interventions for prevention of marijuana use among youth. In: Evidence-Based Resource Guide Series, Preventing Marijuana Use Among Youth. samhsa.gov (2021). p. Pages 45–55. https://store.samhsa.gov/sites/default/files/pep21-06-01-001.pdf

